# Changes in hippocampal inflammatory-related and redox enzyme genes in response to sub-acute restraint stress: Additional dataset

**DOI:** 10.1016/j.dib.2018.11.120

**Published:** 2018-11-28

**Authors:** Hsiao-Jou Cortina Chen, Johnny K. Lee, Tsz Yip, Conrad Sernia, Nickolas A. Lavidis, Jereme G. Spiers

**Affiliations:** a School of Biomedical Sciences, The University of Queensland, St Lucia, Queensland 4072, Australia; b Department of Biochemistry and Genetics, La Trobe Institute for Molecular Science, La Trobe University, Bundoora, Victoria 3083, Australia

**Keywords:** Hippocampus, Neuroinflammatory response, Redox enzymes, Stress

## Abstract

This data article presents complementary results pertaining to the research article entitled “Sub-acute restraint stress progressively increases oxidative/nitrosative stress and inflammatory markers while transiently upregulating antioxidant gene expression in the rat hippocampus” (Chen et al., 2018). The present article provides additional gene expression data of selected neuroinflammatory markers and regulatory enzymes involved in oxidation-reduction reactions. Male Wistar rats aged 7–8 weeks were exposed to control, 1, 2, or 3 episodes of 6-h restraint stress in the light cycle after which the whole brain was quickly removed and the hippocampus excised for relative gene expression analysis. Specifically, mRNA levels of inflammatory regulators including allograft inflammatory factor 1, class II major histocompatibility complex, integrin alpha M, interferon gamma, and prostaglandin-endoperoxide synthase 2 were analyzed by real-time PCR. The gene expression of redox regulatory enzymes including glutathione peroxidase 1, glutathione peroxidase 4, superoxide dismutase 1, superoxide dismutase 2, myeloperoxidase, and NADPH oxidase subunit P47phox were also determined. These data provide useful insights in the molecular basis of inflammatory and redox regulation in the hippocampus following a short term to repeated psychological challenge in rats.

**Specifications table**

**Table:** 

**Subject area**	Neuroscience
**More specific subject area**	Psychoneuroendocrinology and molecular biology
**Type of data**	Figures
**How data was acquired**	Real-time PCR (QuantStudio^TM^ 6 Flex Real-Time PCR System, Applied Biosystems, Foster City, CA)
**Data format**	Analyzed data
**Experimental factors**	Male Wistar rats were randomly allocated into treatment groups of no stress (unstressed), acute (1 Day, single time of 6 h) and repeated (2 and 3 Days, 6 h/day) from 9.00 to 15.00 h using adjustable wire mesh restrainers. At the end of each treatment, whole brain was rapidly removed, and the hippocampus was cryo-dissected for relative gene expression analyses.
**Experimental features**	Total RNA was extracted from each isolated hippocampal tissue, reversed transcribed to cDNA, and the relative expression of targeted genes was determined by real-time PCR.
**Data source location**	School of Biomedical Sciences, The University of Queensland, Brisbane, Australia
**Data accessibility**	The data are available within this article.
**Related research article**	H.J.C. Chen, J.K. Lee, T. Yip, C. Sernia, N.A. Lavidis, J.G. Spiers, Sub-acute restraint stress progressively increases oxidative/nitrosative stress and inflammatory markers while transiently upregulating antioxidant gene expression in the rat hippocampus, Free Radic Biol Med (2018); doi:10.1016/j.freeradbiomed.2018.11.007[1].

**Value of the data**

• Allograft inflammatory factor 1 and integrin alpha M mRNA expression data can be used to demonstrate the regulatory hierarchy of microglial activation markers at the transcriptional level in the hippocampus following repeated stress.
• MHC class II transactivator, interferon gamma, and myeloperoxidase mRNA expression data can be used to indicate stress induces microglial activation.
• These data provide evidence for temporal dynamics of neuroinflammatory and antioxidant regulation following stress.

## 1 Data

**Fig. 1:**
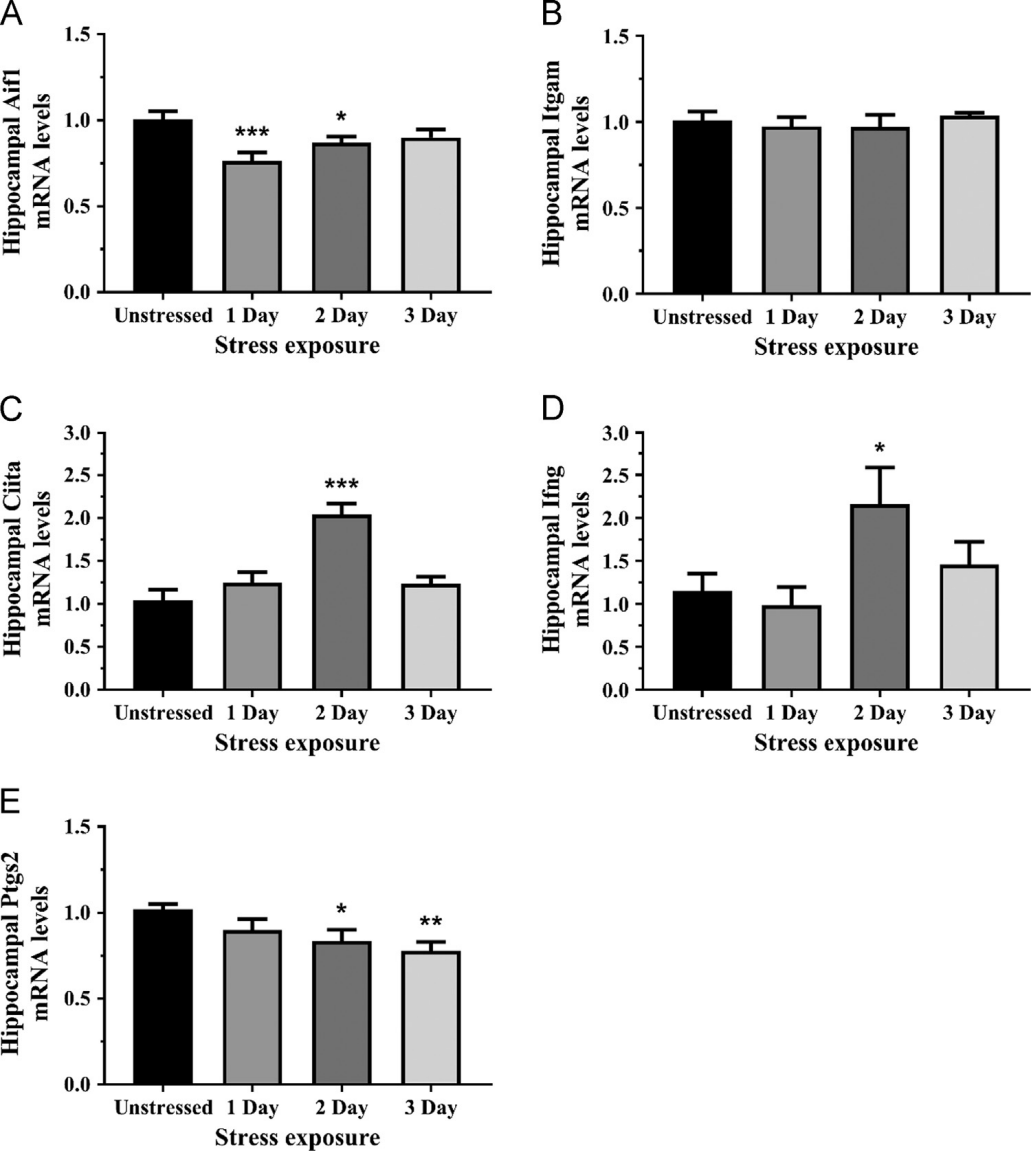
The effects of acute (1 Day, single 6 h) and repeated (2 and 3 Days, 6 h/day) restraint stress on hippocampal (A) allograft inflammatory factor 1 (Aif1; also known as Iba-1), (B) integrin alpha M (Itgam; also known as CD11b), (C) class II, major histocompatibility complex, transactivator (Ciita), (D) interferon gamma (Ifng), and (E) prostaglandin-endoperoxide synthase 2 (Ptgs2; also known as Cox-2) mRNA expression compared to unstressed rats (*n* = 8/group). Result in C was analyzed using non-parametric Kruskal-Wallis test; A, B, D, and E were analyzed using one-way ANOVA with Fisher’s LSD test. Data are expressed as mean ± SEM, *p < 0.05, ** *p* < 0.01, ***p < 0.001.

**Fig. 2:**
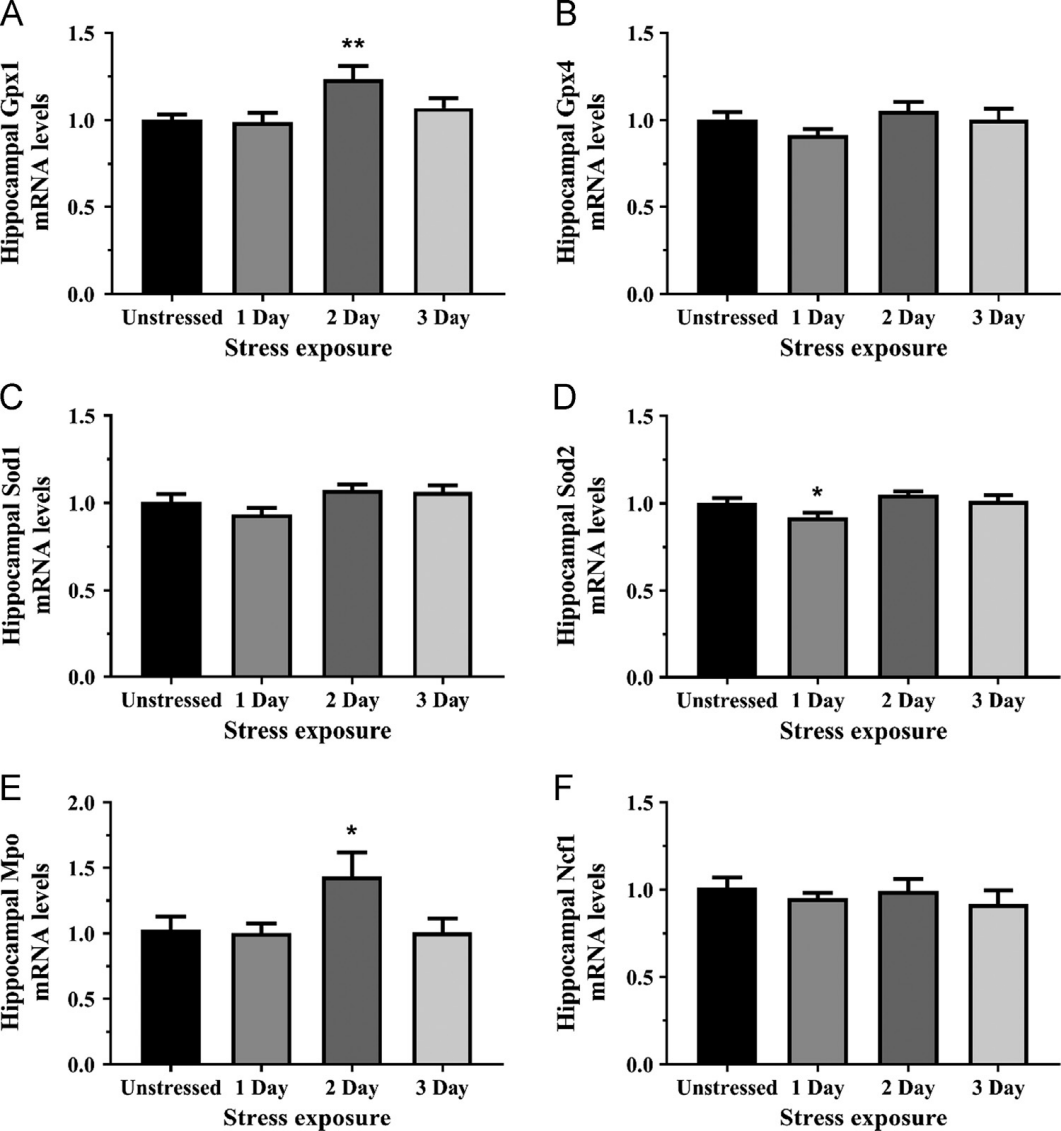
The effects of acute (1 Day, single 6 h) and repeated (2 and 3 Days, 6 h/day) restraint stress on hippocampal (A) glutathione peroxidase 1 (Gpx1), (B) glutathione peroxidase 4 (Gpx4), (C) superoxide dismutase 1 (Sod1), (D) superoxide dismutase 2 (Sod2), (E) myeloperoxidase (Mpo), and (F) neutrophil cytosolic factor 1 (Ncf1; also known as P47phox) mRNA expression compared to unstressed rats (*n* = 8/group. All results were analyzed using one-way ANOVA with Fisher’s LSD test. Data are expressed as mean ± SEM, *p < 0.05 and ** *p* < 0.01.

We have presented genomic data from the hippocampus in support of the research article entitled ‘Sub-acute restraint stress progressively increases oxidative/nitrosative stress and inflammatory markers while transiently upregulating antioxidant gene expression in the rat hippocampus’ published in Free Radical Biology and Medicine [1]. We observed mRNA expression of neuroinflammatory-related markers, allograft inflammatory factor 1 (Aif1l or Iba-1; Fig. 1A), integrin alpha M (Itgam or CD11b; Fig. 1B), major histocompatibility (MHC) class II transactivator (Ciita; Fig. 1C), interferon gamma (Ifng; Fig. 1D), and prostaglandin-endoperoxide synthase 2 (Ptgs2 or Cox-2; Fig. 1E), in the hippocampus following stress treatment. We have also presented genomic data on hippocampal expression of oxidant/anti-oxidant enzymes glutathione peroxidase 1 (Gpx1; Fig. 2A), glutathione peroxidase 4 (Gpx4; Fig. 2B), superoxide dismutase 1 (Sod1; Fig. 2C), superoxide dismutase 2 (Sod2; Fig. 2D), myeloperoxidase (Mpo; Fig. 2E), and neutrophil cytosolic factor 1 (Ncf1 or p47phox; the 47 kDa cytosolic subunit of neutrophil NADPH oxidase; Fig. 2F)

## 2 Experimental design, materials and methods

### 2.1 Experimental animals

The University of Queensland Animal Ethics Committee approved all experimental procedures under approval number SBS/456/14/URG. Individually housed male Wistar rats (Rattus norvegicus) aged 7–8 weeks were sourced from The University of Queensland Biological Resources and maintained within the Australian Institute of Biotechnology and Nanotechnology animal facility. Rats were housed under standard laboratory conditions (22 ± 2 °C; 55 ± 5% humidity) with a 12:12 h light-dark cycle (lights on at 07.00 h) and *ad libitum* access to standard rat chow and water. Prior to experimentation, rats were habituated to human handling for 10 min per day over six days and on each experimental day were transported to an experimental room within the same animal facility for acclimation one hour prior to any experimental procedures.

### 2.2 Experimental protocol

Treatment groups consisted of unstressed, acute (1 Day, single time of 6 h) and repeated (2 and 3 Days, 6 h/day) restraint from 9.00 to 15.00 h (*n* = 8 per group) using restrainers described previously from our laboratory [2]. Control animals were deprived of food and water for the 6-h experimental period. Rats were subsequently overdosed with Pentobarbital Sodium (intraperitoneal injection: Lethabarb, 100 mg/kg, Virbac, Peakhurst, Australia) and the brain was quickly removed and snap-frozen for storage at −80 °C. The hippocampus was isolated from brains sectioned on a cryostat and stored at −80 °C for relative gene expression analyses.

### 2.3 mRNA expression

Total RNA was extracted from hippocampal tissues using the QIAGEN RNeasy mini kit and reverse transcribed into cDNA as described previously [3]. Hippocampal mRNA levels were determined using the Taqman gene expression ‘assay-on-demand^TM^’ assays (Applied Biosystems, Foster City, CA). The primer/probe sets analyzed were FAM-labelled Aif1 (Rn00574125_g1), Ciita (Rn01424725_m1), Gpx1 (Rn00577994_g1), Gpx4 (Rn00820818_g1), Ifng (Rn00594078_m1), Itgam (Rn00709342_m1), Mpo (Rn01460205_m1), Ncf1 (Rn00586945_m1), Ptgs2 (Rn01483828_m1), Sod1 (Rn00566938_m1), and Sod2 (Rn00690588_g1). Each primer/probe was analyzed in reactions multiplexed and normalized with a VIC-labelled primer/probe assay for glyceraldehyde 3-phosphate dehydrogenase (Gapdh; Applied Biosystems, Foster City, CA) and mRNA levels were determined using the formula 2^−^^ΔCT^ where ΔCt = (Ct target gene – Ct Gapdh).

### 2.4 Statistical analysis

Data were analyzed using statistical software GraphPad Prism (Version 7.04, GraphPad Software Inc., San Diego, CA, USA). Data were first analyzed for normality using the Brown-Forsythe test. One-way ANOVA with Fisher׳s least significant difference test were used to compare normally distributed data. A non-parametric Kruskal-Wallis ANOVA with Dunn’s test was used for data with significantly different standard deviations. All comparisons were made against the unstressed group of animals. Results were expressed as mean ± standard error of the mean (± SEM) and *p*-values less than 0.05 were considered statistically significant.
