# Supplementary material for: Changes in hippocampal inflammatory-related and redox enzyme genes in response to sub-acute restraint stress: Additional dataset
Source: Data Brief. 2018 Nov 28;21:2627–32. doi: 10.1016/j.dib.2018.11.120 (PMC6290245; doi:10.1016/j.dib.2018.11.120)

Conflict of Interest and Authorship Conformation Form

- All authors have participated in (a) conception and design, or analysis and interpretation of the data; (b) drafting the article or revising it critically for important intellectual content; and (c) approval of the final version.

- This manuscript has not been submitted to, nor is under review at, another journal or other publishing venue.

- The authors have no affiliation with any organization with a direct or indirect financial interest in the subject matter discussed in the manuscript

Corresponding Author's name	Affiliation
Jereme G Spiers	La Trobe University

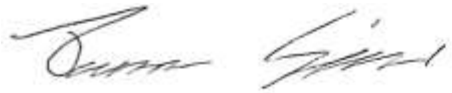

Supplement: Supplementary file 1 — Transparency document [file mmc1.pdf]
